# A Diabetic Emergency One Million Feet Long: Disparities and Burdens of Illness among Diabetic Foot Ulcer Cases within Emergency Departments in the United States, 2006–2010

**DOI:** 10.1371/journal.pone.0134914

**Published:** 2015-08-06

**Authors:** Grant H. Skrepnek, Joseph L. Mills, David G. Armstrong

**Affiliations:** Southern Arizona Limb Salvage Alliance (SALSA), Division of Vascular and Endovascular Surgery, Department of Surgery, University of Arizona College of Medicine, Tucson, Arizona, United States of America; Weill Cornell Medical College in Qatar, QATAR

## Abstract

**Objectives:**

To evaluate the magnitude and impact of diabetic foot ulcers (DFUs) in emergency department (ED) settings from 2006–2010 in the United States (US).

**Methods:**

This cross-sectional study utilized Agency for Healthcare Research and Quality (AHRQ) Healthcare Cost and Utilization Project (HCUP) National Emergency Department Sample (NEDS) discharge records of ED cases among persons ≥18 years with any-listed diagnosis of DFUs. Multivariable analyses were conducted for clinical outcomes of patient disposition from the ED and economic outcomes of charges and lengths of stay based upon patient demographic and socioeconomic factors, hospital characteristics, and comorbid disease states.

**Results:**

Overall, 1,019,861 cases of diabetic foot complications presented to EDs in the US from 2006–2010, comprising 1.9% of the 54.2 million total diabetes cases. The mean patient age was 62.5 years and 59.4% were men. The national bill was $1.9 billion per year in the ED and $8.78 billion per year (US$ 2014) including inpatient charges among the 81.2% of cases that were admitted. Clinical outcomes included mortality in 2.0%, sepsis in 9.6% of cases and amputation in 10.5% (major-minor amputation ratio of 0.46). Multivariable analyses found that those residing in non-urban locations were associated with +51.3%, +14.9%, and +41.4% higher odds of major amputation, minor amputation, and inpatient death, respectively (p<0.05). Medicaid beneficiaries incurred +21.1% and +25.1% higher odds for major or minor amputations, respectively, than Medicare patients (p<0.05). Persons within the lowest income quartile regions were associated with a +38.5% higher odds of major amputation (p<0.05) versus the highest income regions.

**Conclusion:**

Diabetic foot complications exact a substantial clinical and economic toll in acute care settings, particularly among the rural and working poor. Clear opportunities exist to reduce costs and improve outcomes for this systematically-neglected condition by establishing effective practice paradigms for screening, prevention, and coordinated care.

## Introduction

While often initially silent because of attendant neuropathy, lower extremity complications of diabetes constitute a major public health burden in both the developed and developing world.[1,2,3] Overall, these complications frequently develop into diabetic foot ulcers (DFUs) which pose substantial risks of infection and amputation irrespective of critical limb ischemia. The lifetime incidence of DFUs has been estimated to impact 25% of patients, with the condition’s sequelae resulting in serious infections requiring hospitalization among 50% of cases and lower extremity amputation in 25%.[4] The five-year mortality rate associated with DFUs requiring amputation ranges from 39–80%, strikingly similar to the most aggressive forms of cancer.[1,3,5,6] The major risk factors associated with the development of DFUs and subsequent amputation include neuropathy, nephropathy, ischemia (peripheral artery disease or PAD), hypertriglyceridemia, tobacco use, and poor glycemic control.[2,4,7,8]

In addition to the human toll associated with mortality, the direct medical costs of DFUs have also been estimated to constitute one-third of all costs related to diabetes, with two-thirds of these costs incurred within inpatient settings.[5,6] Particularly among working-aged individuals, a two-fold higher likelihood of being admitted through emergency department (ED) settings has been observed.[5] Across numerous disease states, including diabetes, disparities in both the utilization of ED facilities and in health outcomes emanating from these settings have been observed and discussed including age, race or ethnicity, sex, geographic region, socioeconomic status, and insurance coverage.[9–15]

Despite the large clinical and economic burden attributable to DFUs, particularly within acute care settings, only limited research has focused upon ED use for this complex and common condition. Given this research gap, the purpose of the present study was to evaluate the magnitude and impact of cases presenting with a DFU to ED settings from 2006–2010 in the US. More specifically, the objectives were to assess clinical outcomes of patient disposition and economic outcomes of charges and lengths of stay based upon patient demographic and socioeconomic factors, hospital characteristics, and comorbid disease states.

## Methods

Spanning a five-year period for calendar years 2006–2010, this cross-sectional study utilized national emergency department discharge data from the Agency for Healthcare Research and Quality (AHRQ) Healthcare Cost and Utilization Project (HCUP) Nationwide Emergency Department Sample (NEDS).[16] Via a complex sampling design, NEDS data capture patterns of care within approximately one-thousand EDs in the US to permit findings to generalize to over 120 million ED cases per year nationally. As these data are fully-anonymized and do not contain identifiable protected health information, this analysis is designated as exempt from Institutional Review Board Human Subjects Protection.[16]

Inclusion criteria for this study included DFU cases emanating from the ED among adults ≥18 years of age. As no explicit diagnostic code is currently present to identify DFUs, the validated procedure by Sohn et al. (2010) was employed.[17] Therein, ICD-9-CM codes (International Classification of Disease, 9^th^ Edition Clinical Manifestation) of either 707.1x (Ulcer of minor limb, except pressure ulcer) or 707.9 (Chronic ulcer, unspecified) in addition to any diagnosis of diabetes (ICD-9 250.xx) were used.

The key clinical outcomes assessed included patient disposition from the ED, defined as: a) treat-and-release; b) transfer; c) major amputation (i.e., above ankle); d) minor amputation (i.e., involving the foot only); e) inpatient death; and e) inpatient admission. Two economic outcomes were also measured: a) medical-service inflation adjusted charges (US$, 2014) from the ED and inpatient setting, reflecting costs from the perspective of the payer; and b) resource utilization measured by inpatient length of stay (LoS). In more detail, major amputations were defined as ICD-9 84.13–84.19 (i.e., ankle disarticulation, through malleoli of tibia and fibula, below knee, through knee, above knee, hip, abdominopelvic amputation), while minor procedures included ICD-9 84.10–84.12 (i.e., amputation of minor limb, toe, through foot). Independent variables included patient demographics (i.e., age, sex, non-metro/rural residence, regional income quartile level, primary payer), hospital characteristics (i.e., rural location, teaching facility, and geographic region), comorbid case characteristics (i.e., Deyo-Charlson comorbidities, a validated case-mix risk severity measure), the presence of sepsis, and year.[18] Rural categorizations of patient residence and hospital location were defined as micropolitan or non-metropolitan counties according to standardized Urban Influence Codes and National Center for Health Statistics for areas under 50,000 persons. Medicare and Medicaid refer to U.S. federal and state health care coverage programs, respectively.[19] While Medicare predominantly provides coverage to those 65 years of age and older, Medicaid involves state-administered procurement of health care for persons of low income.

Multivariable analyses were used to assess the associations between clinical and economic outcomes and sociodemographic factors, year, hospital characteristics, and comorbid conditions. Patient disposition from the ED was evaluated via a multinomial regression, defining the referent/baseline as an admission-only case.[20,21] Stratified by patient disposition, economic outcomes were analyzed with a generalized linear model (GLM) framework and specified by a gamma distribution and log-link for charges and by a negative binomial distribution using mean dispersions with log-link for LoS.[20] Each of these aforementioned analyses yield coefficients broadly interpreted as relative risks (e.g., <1.0 suggesting a reduced likelihood, = 1.0 suggesting no difference in likelihood, and >1.0 suggesting an increased likelihood). More specifically, the interpretation of these coefficients are as: a) relative risk ratios (RRR) for the multinomial regression of patient disposition from the ED; b) exponentiated beta coefficient estimates (*exp(b)*) for the gamma regression of charges; and c) incidence rate ratios (IRR) for the negative binomial regression of LoS.[20]

All analyses were performed using SAS version 9.2 (Cary, NC) and Stata SE version 12.1 (College Station, TX). An a priori alpha level of 0.05 was used for statistical significance of descriptive statistics and the multinomial regression. Due to the multiple comparisons incurred with analyzing economic outcomes based upon patient disposition, the Simes (1986) procedure was used to control any increase in false discovery rates with the GLM analyses of charges and LoS.[22] Therein, a critical p-value of 0.030 for charges and 0.036 for LoS was calculated. To yield nationally-representative results, a Taylor-series method was used to calculate standard errors based upon HCUP’s weighted sampling design.[15]

## Results

### Descriptives

Across the 2006–2010 time frame in the US, a total of 625.2 million patient cases presented to the ED, of which 8.7% involved any diagnosis of diabetes (n = 54,240,481). Cases with DFUs increased +28.2% from 2006 to 2010 (n = 177,478 to n = 227,439), though consistently averaging 1.9% (n = 1,019,861) of adult ED visits presenting with a diagnosis of diabetes irrespective of year. A majority of DFU cases were admitted as inpatients (81.2%, n = 702,692), ultimately comprising 52.0% of all DFU inpatient cases (n = 1,350,400). Some 34,708 major amputations (i.e., above ankle) were performed in addition to 75,932 minor amputations, representing an overall Major:Minor ratio of 0.457. Death occurred in 2.0% (n = 18,355). The total national bill for DFU cases presenting to the ED summed to $8.78 billion per year (US$, 2014), of which $1.9 billion could specifically be attributed to the ED itself. Therein, treat-and-release cases averaged $2,324 (±4453) in charges, while major amputations involved $115,957 (±112762). While rural, non-urban EDs comprised 12.6% of cases and some 15.5% patients resided in these areas, these regions comprised significantly larger proportions of both treat-and-release cases (Rural ED location = 19.7% and Rural patient residence = 22.1%) and transfers to other acute care facilites (Rural ED location = 38.6% and Rural patient residence = 40.3%) (p<0.05). Concerning case-mix, predominant Deyo-Charlson comorbidities included renal disease, heart failure, peripheral vascular disease, and chronic obstructive pulmonary disease. Sepsis was present in 9.6% of cases overall and was highest among mortality cases (39.0%) and major amputations (30.5%). Table 1 presents the full descriptive statistics of cases stratified by patient disposition and overall.

**Table 1:** Descriptive Statistics of Diabetic Foot Ulcer (DFU) Cases Presenting to Emergency Departments (ED) According to Patient Disposition.

	Treat and Release	Transfer	Major Amputation	Minor Amputation	Inpatient Death	Admission Only	Overall
**Patient/Case Characteristics**							
Mean Age, years	57.4±14.5	65.9±14.9	64.4±13.3	59.0±13.6	72.6±13.2	63.7±14.7	62.5±14.8
Female Sex	38.9%	41.4%	36.1%	29.9%	44.8%	42.2%	40.6%
Micropolitan/Non-Metro Residence	22.1%	40.3%	13.8%	11.5%	14.9%	13.8%	15.5%
Income Region							
1st Quartile (1–24%)	38.1%	36.0%	36.3%	33.4%	27.5%	31.9%	33.2%
2nd Quartile (25–49%)	29.0%	33.9%	27.6%	26.4%	26.9%	26.7%	27.2%
3rd Quartile (50–74%)	20.0%	19.6%	21.1%	22.4%	24.1%	23.0%	22.3%
4th Quartile (75–100%)	13.0%	10.5%	15.0%	17.8%	21.5%	18.4%	17.2%
Primary Payer							
Medicare	44.3%	66.3%	63.3%	45.3%	78.5%	62.0%	58.2%
Medicaid	19.0%	12.6%	15.6%	17.1%	7.4%	14.1%	15.1%
Private Insurance	19.2%	13.2%	13.9%	22.3%	10.5%	16.3%	17.0%
Self-Pay/Underinsured	12.7%	4.4%	4.3%	9.7%	2.0%	4.7%	6.4%
No Charge/Charity	≤1.0%	≤1.0%	≤1.0%	1.3%	≤1.0%	≤1.0%	≤1.0%
Other Payer	3.7%	2.9%	2.5%	4.3%	1.3%	2.4%	2.7%
Year							
2006	17.1%	15.6%	18.1%	16.8%	18.9%	17.5%	17.4%
2007	19.0%	16.8%	20.4%	19.0%	22.2%	19.0%	19.1%
2008	19.3%	17.9%	20.4%	20.2%	21.4%	20.5%	20.2%
2009	20.9%	22.9%	20.9%	20.4%	20.5%	21.0%	21.0%
2010	23.7%	26.8%	20.2	23.5%	72.6±13.2	22.0%	22.3%
**Comorbidities**							
Deyo-Charlson Comorbidity Index	1.0±1.1	1.6±1.4	3.0±1.6	2.4±1.6	3.4±1.9	2.6±1.7	2.3±1.7
Acute Myocardial Infarction	3.5%	5.5%	8.3%	5.9%	18.2%	8.5%	7.6%
Heart Failure	7.3%	18.1%	24.9%	14.7%	49.2%	31.0%	25.6%
Peripheral Vascular Disease	5.6%	13.2%	39.6%	42.8%	18.1%	17.4%	17.9%
Cerebrovascular Disease	≤1.0%	3.6%	7.2%	2.9%	10.6%	6.1%	5.1%
Dementia	≤1.0%	≤1.0%	1.4%	≤1.0%	1.4%	≤1.0%	≤1.0%
COPD	7.2%	10.8%	12.9%	10.3%	24.3%	20.6%	17.2%
Rheumatoid Disease	≤1.0%	≤1.0%	1.1%	≤1.0%	1.7%	2.0%	1.6%
Peptic Ulcer Disease	≤1.0%	≤1.0%	≤1.0%	≤1.0%	1.4%	1.1%	≤1.0%
Mild Liver Disease	≤1.0%	≤1.0%	≤1.0%	≤1.0%	2.9%	1.9%	1.5%
Hemiplegia or Paraplegia	≤1.0%	≤1.0%	≤1.0%	≤1.0%	1.6%	≤1.0%	≤1.0%
Renal Disease	8.3%	17.6%	41.9%	27.1%	44.5%	34.4%	29.6%
Cancer	≤1.0%	1.2%	1.3%	1.3%	5.3%	2.4%	2.0%
Moderate/Severe Liver Disease	≤1.0%	≤1.0%	≤1.0%	≤1.0%	1.9%	≤1.0%	≤1.0%
Metastatic Cancer	≤1.0%	≤1.0%	≤1.0%	≤1.0%	2.7%	≤1.0%	≤1.0%
AIDS	≤1.0%	≤1.0%	≤1.0%	≤1.0%	≤1.0%	≤1.0%	≤1.0%
Sepsis	≤1.0%	2.4%	30.5%	12.3%	39.0%	10.0%	9.6%
**Hospital Characteristics**							
Non-Metro/Rural facility	19.7%	38.6%	8.6%	7.7%	11.0%	11.0%	12.6%
Teaching facility	40.1%	25.5%	52.3%	50.5%	41.5%	44.6%	44.2%
Geographic Location							
Northeast	16.5%	11.2%	21.7%	24.7%	26.8%	24.5%	22.9%
Midwest	22.8%	38.6%	19.7%	18.7%	21.4%	22.1%	22.1%
South	37.1%	28.3%	39.1%	37.7%	34.4%	36.1%	36.3%
West	23.5%	21.9%	19.5%	18.9%	17.3%	17.3%	18.6%
**Economic Outcomes**							
Mean Charges (US$ 2014)	2324±4453	4290±6265	115957±112762	79075±79181	77283±108445	45921±58240	43492±63761
Total Annual Bill (US$ 2014)	0.08 billion	0.01 billion	0.80 billion	1.15 billion	0.28 billion	6.45 billion	8.78 billion
Mean Length of Stay	—	—	16.3±13.2	11.2±8.7	9.0±13.8	7.0±6.9	7.7±8.0
**Sample Size**	176,067	15,262	34,708	72,777	18,355	702,692	1,019,861
	(17.3%)	(1.5%)	(3.4%)	(7.1%)	(1.8%)	(68.9%)	

### Multivariable Analysis: ED Patient Disposition

**Fig 1:**
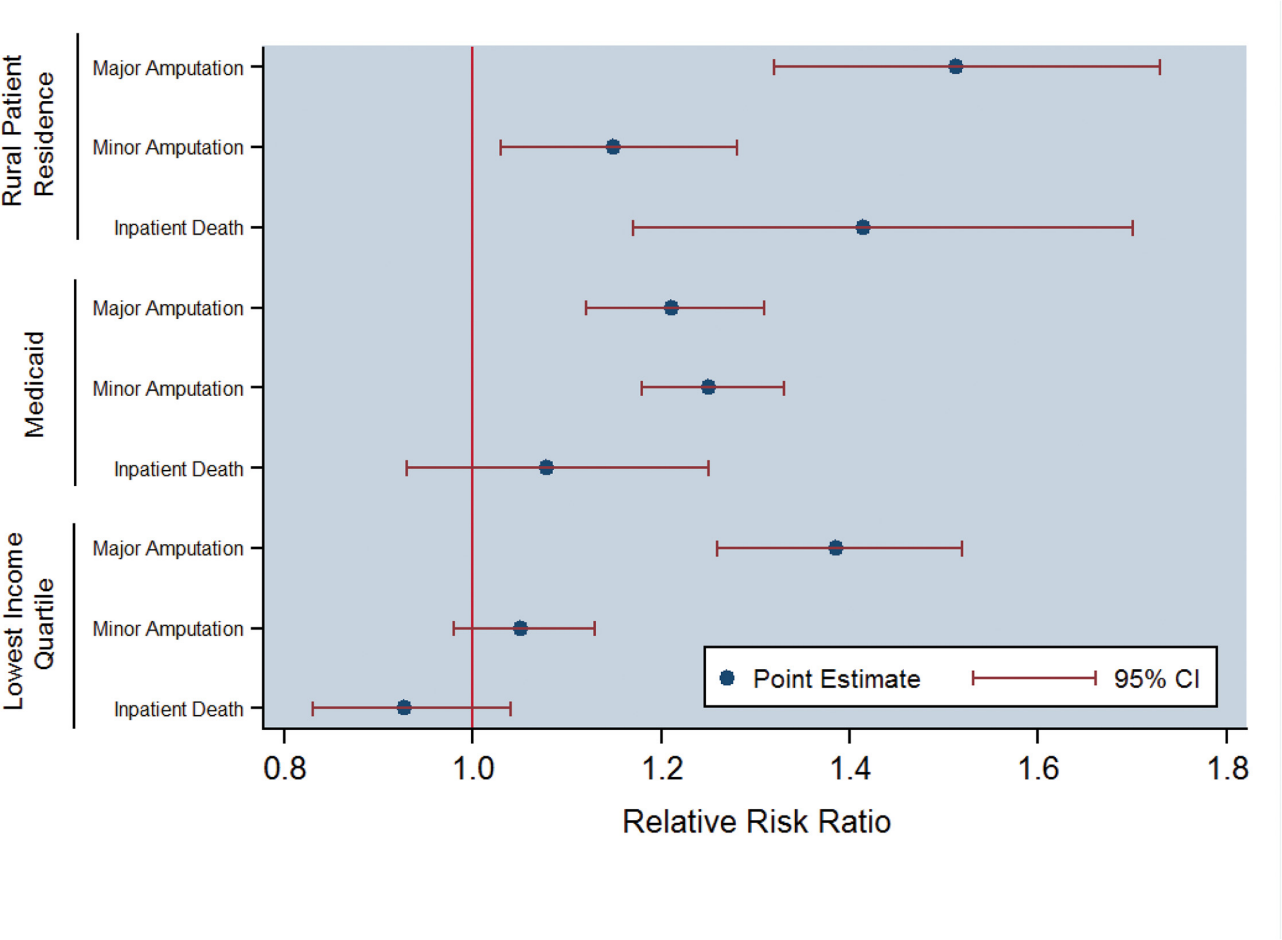
Multivariable-Adjusted Clinical Outcome Disparities Based upon Rural Patient Residence, Medicaid, and Lowest Income Quartile Regions among Diabetic Foot Ulcer Emergency Department Cases the US, 2006–2010. Abbreviations: CI = Confidence Interval. Multinomial regression model controlling for age, sex, residence, regional income quartile, primary payer, calendar year, Deyo-Charlson comorbidities and sepsis, and hospital characteristics of location, teaching facilities, and geographic region (referent case = admission only). Interpretation: Persons residing in rural, non-urban locations were associated with +51.3%, +14.9, and +41.4% higher odds of major amputation, minor amputation, and inpatient death, respectively (p<0.05). While there was no difference in death between Medicare and Medicaid cases presenting to the ED, Medicaid beneficiaries incurred +21.1% and +25.1% higher odds for major or minor amputations, respectively, than Medicare patients (p<0.05). Those residing in lowest income quartile regions were associated with +38.5% higher odds of major amputation versus highest income quartile regions (p<0.05).

Results of the multinomial regression of patient disposition (Table 2, Fig 1) suggested significantly higher odds of poor clinical outcomes particularly based upon patient residence (i.e., rural), regional income level (i.e., lowest income quartile), and primary payer (i.e., Medicaid), graphically presented in Fig 1. To illustrate, relative to admission-only cases, persons residing in small cities or rural areas with populations under 50,000 were associated with +51.3% higher odds of major amputation (OR = 1.513, p<0.001), +14.9% higher odds of minor amputation (OR = 1.149, p<0.001), and +41.4% higher odds of inpatient death (OR = 1.414, p<0.001). Furthermore, those residing in the lowest income quartile region were also associated with a +38.5% higher odds of major amputation (OR = 1.385, p<0.001), while Medicaid beneficiaries incurred a +21.1% and +15.1% higher odds of major amputation (OR = 1.211, p<0.001) and minor amputation (OR = 1.151, p<0.001), respectively.

**Table 2:** Multinomial Regression of Patient Disposition of Diabetic Foot Ulcer (DFU) Cases Presenting to Emergency Departments (ED).

	Treat and Release	Transfer	Major Amputation	Minor Amputation	Inpatient Death	Admission Only
	(*RRR*)	(*RRR*)	(*RRR*)	(*RRR*)	(*RRR*)	[referent]
**Patient Characteristics**						
Age (by decade)	0.917***	1.200***	1.074***	0.900***	1.547***	
Female Sex	0.981	0.906*	0.777***	0.678***	0.976	
Micropolitan/Non-Metro Residence	1.058	1.279*	1.513***	1.149*	1.414***	
Regional Income Level (highest quartile = referent)						
3^rd^ Quartile (50–75%)	1.089	1.201*	1.152**	1.010	1.006	
2^nd^ Quartile (25–49%)	1.223*	1.328**	1.317***	1.024	1.037	
1^st^ Quartile (1–24%), lowest	1.304***	1.317**	1.385***	1.051	0.927	
Primary Payer, Medicare = referent						
Medicaid	1.053	0.971	1.211***	1.251***	1.078	
Private Insurance	1.011	0.774**	0.930	1.447***	0.989	
Self-Pay/Underinsured	1.587***	0.901	1.042	1.815***	1.107	
No Charge/Charity	1.077	1.661	0.603***	1.842***	1.538	
Other	1.028	1.069	1.126	1.726***	0.953	
Year	1.052***	1.120***	0.947***	1.014	0.910***	
**Comorbidities (Deyo-Charlson + Sepsis)**						
Acute Myocardial Infarction	0.628***	0.744**	0.993	0.825***	2.070***	
Heart Failure	0.284***	0.508***	0.736***	0.516***	1.619***	
Peripheral Vascular Disease	0.336***	0.720***	3.016***	3.894***	0.971	
Cerebrovascular Disease	0.201***	0.514***	1.093	0.520***	1.607***	
Dementia	0.251***	0.679	1.229	0.652**	0.762	
Chronic Obstructive Pulmonary Disease	0.382***	0.475***	0.640***	0.570***	1.141**	
Rheumatoid Disease	0.347***	0.375***	0.611***	0.569***	0.996	
Peptic Ulcer Disease	0.135***	0.210***	0.889	0.537***	1.269	
Mild Liver Disease	0.296***	0.571*	0.475***	0.504***	1.567***	
Hemiplegia or Paraplegia	0.282***	0.641	0.951	0.341***	2.089***	
Renal Disease	0.231***	0.448***	1.281***	0.779***	1.378***	
Cancer	0.334***	0.468***	0.578***	0.693***	1.556***	
Moderate-to-Severe Liver Disease	0.255***	0.515	0.626	0.575***	2.325***	
Metastatic Cancer	0.231***	0.474*	0.776	0.558***	2.845***	
AIDS	0.355***	0.556	0.664	0.816	2.648***	
Sepsis	0.019***	0.210***	3.875***	1.266***	6.114***	
**Hospital Characteristics**						
Micropolitan/Non-Metro Location	1.725***	3.132***	0.627***	0.696***	0.751**	
Teaching Facility	1.011	0.736**	1.436***	1.204***	0.943	
Geographic Region (referent = Northeast)						
Midwest	1.536***	3.267***	0.967	0.910*	0.846**	
South	1.295***	1.323*	1.210**	1.036	0.926	
West	1.888***	2.661***	1.384***	1.110*	0.965	

RRR = Relative Risk Ratio

* Statistically significant at p<0.05

** Statistically significant at p<0.01

*** Statistically significant at p<0.001

Over time, significant decreases in the odds of major amputation (OR = 0.947, p<0.001) and inpatient death (OR = 0.910, p<0.001) were observed, although no change in minor amputation was noted (OR = 1.014, p = 0.116). Both treat-and-release cases and transfers were generally associated with less severe comorbid case mixes relative to inpatient admissions, with increased inpatient mortality associated with more severe comorbid case-mixes. Cases presenting with sepsis were at markedly increased odds of inpatient death (OR = 6.114, p<0.001) and major amputation (OR = 3.875, p<0.001).

Concerning hospital characteristics, EDs located within rural communities were significantly more likely to transfer cases to other facilities (OR = 3.132 p<0.001) or offer direct treat-and-release interventions (OR = 1.725, p<0.001), particularly among cases with more advanced case-mix disease severities or sepsis. Therein, a decreased odds of approximately −25% or more was observed in these facilities concerning either major amputation (OR = 0.627, p<0.001), minor amputation (OR = 0.696, p<0.001), or inpatient death (OR = 0.751, p = 0.009). Teaching facilities were significantly less likely to transfer patients (OR = 0.736, p = 0.002) and were more likely to perform either major amputations (OR = 1.436, p<0.001) or minor amputations (OR = 1.204, p<0.001). Despite this, no difference in inpatient mortality was observed among teaching versus non-teaching EDs (OR = 0.943, p = 0.192). Broad differences were also observed based upon geographic region, with generally higher odds of treat-and-release or transfers occurring in areas beyond the Northeast, higher odds of major amputation in the South and West, and higher odds of minor amputation in Western regions (p<0.05).

### Multivariable Analysis: Economic Outcomes

The multivariable analysis of economic outcomes for charges (Table 3) and LoS (Table 4) indicated significant increased annual inflation-adjusted charges over time for treat-and-release and transfer cases of slightly over +16%, suggesting a potentially greater intensity of care offered among these cases. While several comorbid conditions were associated with increased charges or LoS, only renal disease and sepsis were consistently associated with both higher charges and longer LoS (p<0.001) irrespective of patient disposition. Even though LoS decreased annually (p<0.001) among cases involving any inpatient admission (i.e., major or minor amputation, inpatient death, admission only), inflation-adjusted charges did not significantly change in these groups.

**Table 3:** Gamma Regression of Total Charges Among Diabetic Foot Ulcer (DFU) Cases Presenting to Emergency Departments (ED) According to Patient Disposition.

	Treat and Release	Transfer	Major Amputation	Minor Amputation	Inpatient Death	Admission Only
	[*exp(b)*]	[*exp(b)*]	[*exp(b)*]	[*exp(b)*]	[*exp(b)*]	[*exp(b)*]
**Patient Characteristics**						
Age (by decade)	1.020	1.014	0.961	1.048***	0.877***	0.993
Female Sex	0.992	1.023	1.043	1.043***	0.972	1.014***
Micropolitan/Non-Metro Residence	1.005	0.883	0.842	0.892***	1.054	0.907***
Regional Income Level (highest quartile = referent)						
3^rd^ Quartile (50–75%)	1.099	1.162	1.014	1.064	1.054	0.988
2^nd^ Quartile (25–49%)	1.107	1.199	0.991	1.032	0.943	1.022
1^st^ Quartile (1–24%), lowest	1.111	1.139	1.036	1.048	0.960	1.038
Primary Payer, Medicare = referent						
Medicaid	0.966	0.899	1.117	1.140***	1.383***	1.037***
Private Insurance	1.028	1.136	0.994	0.942***	0.895	0.958***
Self-Pay/Underinsured	0.891***	0.882	1.109	1.066	1.109	0.993
No Charge/Charity	1.036	0.676	1.470	0.973	1.818	1.066
Other	0.824	0.818	1.100	1.199***	0.780	1.040
Year	1.164***	1.169***	0.994	0.988	0.968	0.997
**Comorbidities (Deyo-Charlson, Sepsis)**						
Acute Myocardial Infarction	1.336***	0.810	1.097***	1.169***	1.073	1.149***
Heart Failure	1.362***	1.243***	1.141***	1.225***	0.995	1.144***
Peripheral Vascular Disease	1.408***	1.214***	0.925***	0.974	0.818***	1.033***
Cerebrovascular Disease	2.349***	1.550***	0.954	1.234***	1.008	1.099***
Dementia	1.417	0.701	0.838***	0.734***	0.919	0.921***
Chronic Obstructive Pulmonary Disease	1.320***	1.125	0.995***	1.089***	1.044	1.044***
Rheumatoid Disease	1.416***	0.875	0.804	0.907	0.692***	0.916***
Peptic Ulcer Disease	2.137***	1.186	1.172	1.241***	1.592***	1.240***
Mild Liver Disease	1.434***	0.906	1.132	0.856	0.802	0.970
Hemiplegia or Paraplegia	1.712***	1.083	0.940	1.077	1.194	1.195***
Renal Disease	1.504***	1.220***	1.162***	1.216***	1.137***	1.130***
Cancer	1.458***	1.335	1.108	1.124	1.011	1.143***
Moderate-to-Severe Liver Disease	1.535	0.931	0.976	1.133	0.942	1.133***
Metastatic Cancer	1.086	0.929	1.016	1.338	0.851	1.096
AIDS	1.089	0.876	0.959	1.106	0.562	1.063***
Sepsis	3.650***	1.393***	1.383***	1.445***	1.324***	1.681***
**Hospital Characteristics**						
Micropolitan/Non-Metro Location	0.779***	0.876	0.911	0.661***	0.503***	0.608***
Teaching Facility	1.072	0.127	1.024	0.832	1.085	0.988
Geographic Region (referent = Northeast)						
Midwest	1.326***	1.061	0.696	0.669***	0.670***	0.685***
South	1.465***	1.516***	0.834	0.832***	0.912	0.841***
West	0.293***	0.364***	1.181	1.057	1.325***	1.142

*exp(b)* = exponentiated beta coefficient

***Statistically significant below the computed Simes (1986) false discovery rate p-value (p<0.030)

**Table 4:** Negative Binomial Regression of Inpatient Length of Stay (LoS) of Diabetic Foot Ulcer (DFU) Cases Presenting to Emergency Departments (ED) According to Patient Disposition.

	Major Amputation	Minor Amputation	Inpatient Death	Admission Only
	[*IRR*]	[*IRR*]	[*IRR*]	[*IRR*]
**Patient Characteristics**				
Age (by decade)	0.981***	1.035***	0.920***	1.010***
Female Sex	1.050***	1.032***	1.078	1.042***
Micropolitan/Non-Metro Residence	0.956	0.993	1.001	0.986
Regional Income Level (4th quartile, highest = referent)				
3^rd^ Quartile (50–75%)	0.998	1.046	0.948	1.006
2^nd^ Quartile (25–49%)	0.955	1.030	1.054	1.032***
1^st^ Quartile (1–24%), lowest	1.001	1.036	1.039	1.048
Primary Payer, Medicare = referent				
Medicaid	1.160***	1.234***	1.505***	1.099***
Private Insurance	1.025	0.972	0.979	0.957***
Self-Pay/Underinsured	1.153***	1.110***	1.001	1.011
No Charge/Charity	1.625***	1.107	1.811***	1.153
Other	1.113	1.197***	0.921	1.097***
Year	0.966***	0.956***	0.935***	0.971***
**Comorbidities (Deyo-Charlson + Sepsis)**				
Acute Myocardial Infarction	1.048	1.053***	0.846***	0.964***
Heart Failure	1.136***	1.186***	1.095	1.110***
Peripheral Vascular Disease	0.949***	1.008	0.928	1.004
Cerebrovascular Disease	0.992	1.172***	0.929	1.051***
Dementia	0.805***	0.859***	0.952	1.056***
Chronic Obstructive Pulmonary Disease	0.945***	1.059***	1.001	1.004
Rheumatoid Disease	0.817***	0.993	0.745***	0.957***
Peptic Ulcer Disease	1.118	1.298***	1.374***	1.138***
Mild Liver Disease	1.052	0.993	0.925	1.012
Hemiplegia or Paraplegia	0.931	1.141	0.924	1.167***
Renal Disease	1.114***	1.147***	1.126***	1.077***
Cancer	1.133	1.121***	1.100	1.120***
Moderate-to-Severe Liver Disease	0.965	1.288	1.033	1.118***
Metastatic Cancer	0.998	1.154	0.958	1.094***
AIDS	0.902	0.929	0.331***	0.976
Sepsis	1.257***	1.379***	1.207***	1.572***
**Hospital Characteristics**				
Micropolitan/Non-Metro Location	1.066	0.917***	0.855	0.840***
Teaching Facility	1.075***	1.033	1.159***	1.013
Geographic Region (referent = Northeast)				
Midwest	0.742***	0.719***	0.609***	0.768***
South	0.884***	0.847***	0.839***	0.884***
West	0.847***	0.781***	0.805***	0.819***

IRR = Incidence Rate Ratio

***Statistically significant below the computed Simes (1986) false discovery rate p-value (p<0.036)

Concerning the association between economic outcomes and sociodemographic characteristics, higher charges were observed among Medicaid recipients versus Medicare patients, including +14.0% for lower amputations (*exp(b)* = 1.140, p<0.001), +38.3% for inpatient mortality (*exp(b)* = 1.383, p = 0.003), and +3.7% for inpatient admissions (*exp(b)* = 1.037, p = 0.021). Medicaid beneficiaries incurred longer lengths of stay, ranging from +9.9% higher for admission-only cases (IRR = 1.099, p = 0.001) to +50.5% higher for mortality cases (IRR = 1.505, p = 0.006). Females had slightly higher charges and LoS than males for lower amputations (*exp(b)* = 1.043, p = 0.013 and IRR = 1.032, p = 0.018). Geographically, shorter lengths of stay were found across every region versus the Northeast (p<0.001), and charges varied according to region and patient disposition.

## Discussion

This investigation of over one million ED cases presenting with DFUs suggests that diabetic foot complications remain common, complex, and costly. The national inpatient and ED bill summed to $8.78 billion per year, averaging $115,957 per case for major amputations (US$ 2014). This corresponds, at least in magnitude, to estimates reported by Kerr, Rayman, and Jeffcoate (2014) of $1 billion for inpatient and outpatient care to the National Health Service in England.[23] Importantly, significant disparities in health outcomes were observed across numerous patient demographics, clinical comorbidities, and ED characteristics. Persons residing in rural areas (i.e., under 50,000 people) were associated with higher adjusted odds of major amputation, minor amputation, and inpatient death of +51.3%, +14.9%, and +41.4%, respectively (p<0.05). An increased odds of major amputation was also independently observed among Medicaid recipients (+21.1% versus Medicare) and lowest income quartile regions (+38.5% versus highest quartile regions). Building upon the IOM (2002) report “Unequal Treatment: Confronting Racial and Ethnic Disparities in Health Care”, Richards and Lowe (2003) summarized findings from the Consensus Conference on Disparities in Emergency Health Care, noting that a disproportionately greater burden of illness has been frequently observed among persons living in rural areas and the working poor in ED settings.[9,24] To mitigate these, and other, differences in health outcomes, the review of health care interventions targeting disparities in diabetes by Peek, Cargill, and Huang (2007) recommended utilizing culturally-tailored programs, providing feedback and education to clinicians, and developing team-based health delivery models.[25]. This may be especially important in care of the diabetic foot, worldwide, as actual costs may not be reflective of income level. For example, Cavanagh et al. (2012) reported in several case vignettes that the cost to heal a complex diabetic foot wound ranged from 3 months’ wages in Chile, to 10 months’ wages in the USA, to 5.7 years wages in India.[26]

Though not relating specifically to DFUs, Menchine, Wiechmann, Peters, and Arora (2012) analyzed 20.2 million diabetes cases presenting to the ED from 1997–2007 via the National Hospital Ambulatory Medical Care Survey (NHAMCS), constituting 1.7% of all ED cases nationally.[15] While a significant +5.6% annual increase in the proportion of diabetes-related cases was reported (p<0.05), this observation was attributed to a higher prevalence of diabetes across the overall population. Importantly, an increased odds (p<0.05) of diabetes-related visits was found to be associated with Medicaid or Medicare health care coverage versus private insurance (OR_Medicare_ = 1.82, OR_Medicaid_ = 1.74), black or Asian race versus white (OR_Black_ = 1.84, OR_Asian_ = 1.32), Hispanic ethnicity versus non-Hispanic (OR_Hispanic_ = 1.60), and increasing age. Ginde, Espinola, and Camargo (2008) also reported that diabetes-related ED visits involving hypoglycemia accounted for approximately 380,000 ED visits per year from the 1993–2005 NHAMCS (National Hospital Ambulatory Medical Care Survey), with visit rates being higher among females, blacks, and Hispanics versus males, whites, or non-Hispanics (p<0.001).[14] Disparities were observed based on region and age, including higher visit rates in the South and MidWest versus the West (p<0.001) and among both younger persons between 0–44 years of age and older persons age 75 years and above versus those from 45–75 years (p<0.001). Gaskin et al. (2013) more recently reported that irrespective of race, individual poverty and living in poor neighborhoods were associated with higher odds of having diabetes.[27] Therein, concentrated poverty may create marked barriers to health services and barriers to healthy lifestyles, among other concerns. In earlier work, among almost nine thousand persons with diabetes in a large staff-model health maintenance organization from 1993–1995, Ramsey et al. (1999) reported a cumulative index of DFUs of 5.8%, culminating in a two-year post-diagnosis attributable cost with DFUs of $27,987 ($30385 per year, US$ 2014) versus the average single case presenting to the ED in the present study of $43,492 (US$ 2014).[28] Some 15.6% required amputations across the entire three years of Ramsey et al. (1999) study versus the single presentation of 10.5% observed in the current work. Relating to these studies, findings from the current investigation suggest that the rate of DFU cases was relatively constant as a percentage of diabetes ED cases and of overall ED cases at 1.9% and 0.2% per year and that rural ED facilities were more likely to either treat-and-release cases or to transfer them, presumably to tertiary care units. Independent differences concerning a patient’s disposition/outcomes from the ED were also observed according to demographics (i.e., age, sex, rural residence, regional income quartile), clinical comorbidities (i.e., Deyo-Charlson comorbidities and sepsis), and ED characteristics (i.e., rural location, teaching facilities, geographic region).

Concerning the likelihood of utilizing the ED among patients with and without diabetes, Egede (2004) utilized the Center for Disease Control (CDC) National Health Interview Survey (NHIS) and found that that no significant differences existed in the odds of ED use between those with and without diabetes in the year 1999 after controlling for self-reported data on demographics and socioeconomic status, seven various comorbid conditions, perceived health status, and selected diabetes-related complications (i.e., coronary artery disease, stroke, end-stage renal failure, macular degeneration, retinopathy, blindness).[13] Among individuals with diabetes, however, significant correlates of multiple ED visits (p<0.05) included unemployment, younger age, having a single usual source of care, perceived worsening of health, three or more comorbidities, and presence of diabetes complications. While the current study did not assess the odds of ED use, patient disposition from the ED was observed to vary according to other variables not captured in Engde (1999), including patient sociodemographics (e.g., residence, regional income quartile), primary payer (e.g., Medicaid), additional comorbid conditions (e.g., sepsis, Deyo-Charlson), hospital characteristics (e.g., rural), and calendar year.

In observing that two-thirds of diabetes-related ED cases were directly discharged, Menchine et al. (2012) emphasized that outcomes in diabetes were highly contingent upon care delivered within ambulatory settings and that several ED visits may have been averted through improved preventive care and disease management programs.[15] Washington, Andrews, and Mutter (2013) also stressed that ED use among diabetics is likely multifaceted and related to the presence of complications, poor adherence to treatment and lifestyle modification plans, and lack of primary care.[29] The current study’s findings that 81.2% of ED cases with DFUs were admitted and only 17.3% were directly treated-and-released suggests presentation of individuals with an increased severity of illness warranting more intensive care, albeit also potentially preventable with improved access to ambulatory care management programs.

While nationally-representative data for ED cases to assess patient disposition and economic outcomes among DFU cases was used in the present work, certain limitations should be addressed. Foremost, although key variables known to be associated with outcomes were included, unmeasurable or exogenous factors beyond these discharge data may be relevant in the regression model’s specification. Clinical information was not present to classify the extent of wounds, degree of ischemia, or severity of infection, all factors highly likely to impact costs and outcomes.[30] Additionally, given that the unit of analysis was the presentation of a case to the ED and not the patient himself over a specified time period, revisits or readmissions could not be reported, nor could the role of treatment within ambulatory care settings. The validated method used as a case definition for DFUs does not suggest that this complication was necessarily the principal diagnosis on record. Finally, generalizing findings specifically to individual patients or hospital systems or patients should be undertaken with caution.

Overall, the interpretation of disparities in health care remains complex, particularly given that root causes are often inadequately measured or addressed.[9,24] Future research must continue to seek robust assessments of health delivery infrastructure, barriers to care, perceptions of providers, and preferences among treatment interventions.[31–35] Among those with diabetes, prior research has suggested that a marked underdiagnosis of complications is present in rural locations, with several barriers present: 1) a lower likelihood of receiving professional foot examinations; 2) inadequate practice of conducting a self-foot examination; and 3) a lack of communication across health care providers to engage patients in diabetes self-management education programs.[36] Diabetes-related lower limb amputations are associated with considerable morbidity and mortality and are usually preceded by foot ulceration.[37] Recognizing the potential for severe morbidity related to foot ulcers, many international programs advocate the widespread establishment and implementation of preventive foot care programs with significant emphasis on patient education.[38] Currently, annual assessment procedures are recommended to identify patients with diabetes who are at risk of foot ulceration.[39] Data consistently suggest that patients who have seen a foot specialist along with another member of the diabetes care team in the preceding year have up to an 64–84% lower risk of undergoing an amputation in the subsequent six years.[40,41] Additionally, data suggest that removal of preventive foot services on a statewide basis increases hospitalization by 38% with an increase in amputation, sepsis and death increasing by some 49%.[42]

## Conclusion

Diabetic foot complications exact a substantial toll on resource utilization as a percentage of overall diabetes care provided in the ED. Development of better systems of urban and rural comprehensive outpatient diabetic foot services to provide earlier coordinated care for this frequently silent condition, should be a major point of healthcare emphasis as it has the potential to reduce costs of emergency care and improve outcomes.
